# The effects of lexical frequency and homophone neighborhood density on incomplete tonal neutralization

**DOI:** 10.3389/fpsyg.2022.867353

**Published:** 2022-11-24

**Authors:** Yifei Bi, Yiya Chen

**Affiliations:** ^1^College of Foreign Languages, University of Shanghai for Science and Technology, Shanghai, China; ^2^Leiden University Centre for Linguistics, Leiden, Netherlands; ^3^Leiden Institute for Brain and Cognition, Leiden, Netherlands

**Keywords:** lexical frequency, homophone neighborhood density, tonal neutralization and merger, sound change, speech production, Dalian Mandarin

## Abstract

We investigated the effects of lexical frequency and homophone neighborhood density on the acoustic realization of two neutralizing falling tones in Dalian Mandarin Chinese. Monosyllabic morphemes containing the target tones (Tone 1 and Tone 4) were produced by 60 native speakers from two generations (middle-aged vs. young). The duration of tone-bearing syllable rhymes, as well as the F0 curves and velocity profiles of the lexical tones were quantitatively analyzed *via* linear mixed-effects modeling and functional data analysis. Results showed no durational difference between T1 and T4. However, the F0 contours of the two falling tones were incompletely neutralized for both young and middle-aged speakers. Lexical frequency showed little effect on the incomplete tonal neutralization; there were significant differences in the turning point of the two falling tones in syllables with both high and low lexical frequency. However, homophone neighborhood density showed an effect on the incomplete neutralization between the two falling tones, reflected in significant differences in the slope and turning point of the F0 velocity profiles between the two tones carried by syllables with low density but not with high density. Moreover, homophone neighborhood density also affected the duration, the turning point of F0 curves, and velocity profiles of the T1- and T4-syllables. These results are discussed with consideration of social phonetic variations, the theory of Hypo- and Hyper-articulation (H&H), the Neighborhood Activation Model, and communication-based information-theoretic accounts. Collectively, these results broaden our understanding of the effects that lexical properties have on the acoustic details of lexical tone production and tonal sound changes.

## Introduction

The notion of neutralization presupposes the concept of contrast. Neutralization refers to sound change where a contrast that exists in a language is lost in some particular contexts in its synchronic grammar [see reviews in Yu (2011), Kubozono and Giriko (2018), and references therein]. Existing acoustic studies on neutralization have explored various factors ranging from lexical properties (e.g., word frequency and orthography) to speaker characteristics (e.g., geographical locations of the population and speaking style) (Warner et al., 2004, 2006; Kharlamov, 2014; Roettger et al., 2014; Braver and Kawahara, 2016; Nicenboim et al., 2018; Kong and Shengyi, 2019).

One interesting area of neutralization research examines the acoustic characteristic of constant devoicing. These studies focused on Indo-European languages, such as Dutch (Warner et al., 2004, 2006), German (Roettger et al., 2014; Nicenboim et al., 2018), Catalan (Dinnsen and Charles-Luce, 1984), and Russian (Kharlamov, 2014; Matsui et al., 2017). For example, Warner et al. (2004) compared the acoustic realization of both long and short vowels before the Dutch alveolar /t/ and /d/ coda. They showed that vowels preceding an underlying /d/ were significantly longer than those preceding an underlying /t/. Furthermore, following long vowels, underlying /t/ had a longer burst duration than underlying /d/. Their study exemplifies how the so-called neutralized consonants (based on impressionistic observation) may nevertheless exhibit reliable acoustic differences and show characteristics of incomplete neutralization with data elicited with a controlled experimental design.

Studies have looked at a range of factors that can possibly encourage incomplete neutralized voicing contrasts in speakers. For example, orthography has been argued to bias speakers to “artificially” hyper-articulate the /t/ vs. /d/ contrast given their different written forms (e.g., Fourakis and Iverson, 1984; Warner et al., 2004). Another commonly discussed factor is the voicing contrasts of the stimuli in the language, which presumably motivates the speakers to preserve the underlying voicing distinction between minimal pairs (in comparison to non-minimal pairs) (Port and O’Dell, 1985; Ernestus and Baayen, 2006; Kulikov, 2012). Kharlamov (2014) examined the role of orthography, phonology, and elicitation tasks (reading vs. picture naming) in the acoustic realization of Russian voicing contrasts and argued that these factors influence the incomplete neutralization of Russian voicing contrasts through different acoustic parameters. Matsui et al. (2017) further showed the importance of controlling the lexical frequency and minimal-pair effects in investigating incomplete neutralization in Russian. Despite the increasing number of studies on incomplete voicing neutralization, there is still a lack of consensus on how exactly different factors condition neutralization and a lack of research that directly examines lexical effects such as phonological neighborhood density and lexical frequency on (incomplete) voicing neutralization.

Compared with the body of quantitative studies on segmental voicing neutralization in a wide range of (Indo-European) languages, much less research has focused on neutralization at the suprasegmental level, such as lexical tones. A handful of studies have investigated lexical tonal neutralization in Cantonese (e.g., Bauer et al., 2003; Mok et al., 2013; Cheng, 2017; Liang, 2018; Lin et al., 2021). A number of studies have investigated the status of tonal contrast between two merging tonal pairs. Further research has been conducted on Standard Chinese, where researchers have investigated possible neutralization of the lexical Rising tone (LR) with the sandhi rising variant (SR) of the Low tone, which is realized with a comparable rising F0 contour as the lexical Rising tone when preceding another Low tone (Chen and Yuan, 2007; Cheng et al., 2013; Yuan and Chen, 2014; Li and Chen, 2015; Nixon et al., 2015; Lin and Hsu, 2018; Politzer-Ahles et al., 2019).

Only a few studies have investigated the possible effects of lexical properties such as word frequency on tonal neutralization/merger. For example, Yuan and Chen (2014) explored the acoustic characteristics of the LR and SR in telephone conversations and broadcast news speech. They found SR is different from LR in terms of the magnitude of the F0 rise and the time span of the F0 rise. Furthermore, they discovered that SR in the most highly frequent words (>1,000 in frequency counts of 3,431,707 words in the Xinhua newswire) showed a greater difference from the LR than in less-frequent words. Furthermore, Mok et al. (2013) investigated the effect of word (token) frequency on Cantonese tone merger. Slight differences were shown in the tone merger between high-frequency and low-frequency words. Finally, Kong and Shengyi (2019) studied the effect of frequency on tonal reduction in Standard Chinese. Their results showed that the acoustic characteristic of reduction-induced neutralized tones (and the tone-carrying syllables) correlates directly with lexical frequency. However, much is still to be understood concerning how different factors condition tonal neutralization.

In this study, we aimed to shed light on the role of lexical properties in a sound-change-related process of tonal (incomplete) neutralization. There has been increasing interest in studying the effects of lexical properties on speech production. For example, one widely studied lexical property is word frequency. High-frequency words are typically produced with a shorter duration, reduced vowels, and reduced pitch range than low-frequency words (e.g., Pluymaekers et al., 2005; Zhao and Jurafsky, 2007; Mousikou and Rastle, 2015). Another key lexical property is phonological neighborhood density. Words from high (dense) neighborhood density are typically produced faster, more accurately, and hyper-articulated compared with words from low (sparse) neighborhoods (e.g., Munson and Solomon, 2004; Wright, 2004; Baus et al., 2008; Gahl, 2008; Dell and Gordon, 2011; Scarborough, 2013; Gahl and Strand, 2016).

It is essential to note that with studies based mainly on Indo-European languages, the phonological neighborhood is typically defined with the one-phoneme difference rule. Phonological neighbors are two words that differ in only one phoneme by substitution, deletion, or addition (Luce and Pisoni, 1998; Luce et al., 2000; Vitevitch and Luce, 2016). Kapatsinski (2006) proposed a new method and defined words as phonological neighbors if they share at least two-thirds of their total segmental string. This draws upon evidence from lexical decision tasks, naming reaction times, and familiarity rating.

Yao and Sharma (2017) have proposed that in tonal languages, such as Mandarin, to define phonological neighbors by the one-phoneme/tone difference rule: any two syllables that only differ in one phoneme or tone are phonological neighbors. Given the abundant Mandarin homophones (i.e., monosyllabic morphemes with the same segmental syllables and tone), psycholinguistic studies on Chinese spoken word production/recognition often employ the notion of homophone neighbors (e.g., Chen et al., 2009, 2016; Wang et al., 2012), including only words that share both segments and tone. This study follows that tradition and defines neighborhood as homophone neighborhood, with homophones sharing the same segmental syllable and lexical tone. The empirical base of our investigation is Dalian Mandarin given the reported ongoing sound change and (incomplete) neutralization concerning two lexical tones.

### Dalian Mandarin

Dalian Mandarin is a dialect of Mandarin, mainly spoken in the urban areas of Dalian City in Northeast China, about 460 km from the capital Beijing. Dalian Mandarin belongs to the Jiao-Liao Mandarin dialect group, a major Sinitic Mandarin group. Song (1963) states that Dalian Mandarin has four lexical tones produced in isolation: T1 has a falling and slight rising F0 contour (312), T2 a rising F0 contour (34), T3 a dipping contour (213), and T4 a falling contour (53). Here, the numerical numbers represent the pitch levels/ranges, following Chao’s pitch annotation system (Chao, 1968), where 1 refers to the lowest end of a speaker’s pitch range, and 5 is the highest end.

Sound changes have been reported in T1 and T4 (Gao, 2007; Liu, 2009), and they are both realized with a high-falling F0 contour. Gao (2007) conducted an acoustic analysis of data data collected from three generations of (young: aged below 29, middle: aged from 50 to 59, and old: aged from 70 to 80). The results showed that the old-generation speakers produced a different citation form of T1 (411 rather than 312) and T4 (52/51 instead of 53). However, for the middle and young-generation speakers, T1 and T4 have become even closer and are transcribed to share the citation form (51). Liu (2009) also concluded that in present-day Dalian Mandarin, T1 is now a high-falling tone (51).

**FIGURE 1:**
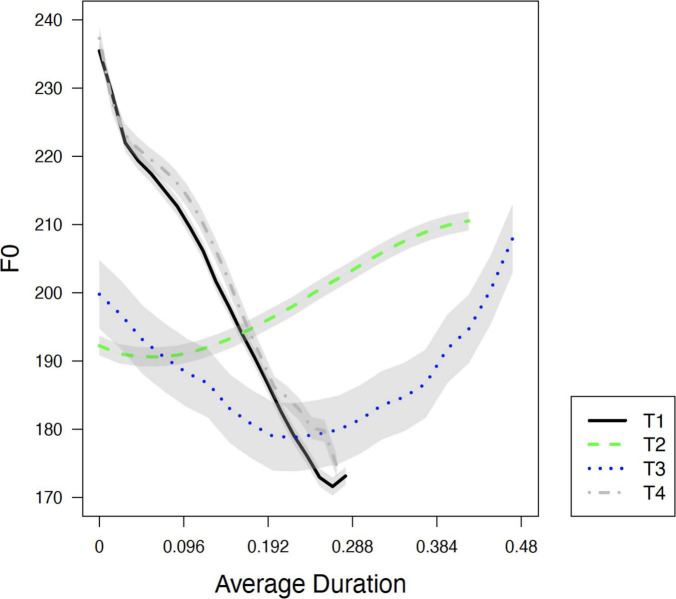
Four lexical tones in present-day Dalian Mandarin produced in isolation, with each tonal contour based on 20 samples produced by a young male native speaker. Lines represent the mean. Shaded areas stand for the standard error of the mean (±1.3 for T1, ±1.4 for T2, ±5 for T3, and ±1.6 for T4).

Figure 1 plots the average F0 contours of the four lexical tones, with each tonal contour based on 20 samples produced by a young male native speaker (born in 1990). T2 has a rising F0 contour (35) and T3 a dipping contour (213). T1 and T4 are both realized with a falling contour (51), although it is not clear to what extent they have merged.

### The current study

The current work was inspired by two notable observations. First, no quantitative analysis has been conducted on whether T1 and T4 in Dalian Mandarin are completely neutralized. Regardless, impressionistically, they seem to have merged to have the same tonal identity, but likely remained incompletely neutralized. This possibility is then similar to the incomplete voicing neutralization in many Indo-European languages. If this was the case, detailed acoustic analyses and proper statistical analyses of data produced by a sufficiently large number of participants are essential for us to detect the potentially subtle tonal differences. Second, previous Dalian Mandarin studies only elicited a limited set of frequent words [i.e., the so-called vocabulary of daily uses in Gao (2007)]. We know that factors such as different lexical frequencies and phonological neighborhood density could significantly affect the retrieval and production of spoken words. These factors could also be further conditioned by speakers’ age [e.g., Gordon and Kurczek (2014) on the diminished facilitation effect of neighborhood density due to aging]. Given the potential ongoing changes of these two tones, speaker age is likely to have a substantial impact on tonal realizations. The goal of this study, therefore, aimed to investigate the possible impact of speakers’ age, lexical frequency, and homophone phonological neighborhood density on the (in)complete neutralization of T1 and T4 in Dalian Mandarin. Specifically, the following questions will be addressed: (1) Are the two falling tones neutralized for middle-aged and young-generation speakers of Dalian Mandarin? (2) How do lexical frequency and homophone neighborhood density affect tonal realization and neutralization?

## Materials and methods

### Participants

30 middle-aged (mean age: 50; SD: 3.6) and 30 young (mean age: 22; SD: 3.6) native speakers of Dalian Mandarin participated in the experiment. The participants were selected from the urban area of Dalian City, including the districts of Sha Hekou, Zhong Shan, Xi Gang, and Gan Jingzi, and self-reported to have normal vision and no history of speech disorders. Informed consent was obtained from all participants before beginning the experiment, and all participants were paid to take part.

### Materials

The target stimuli included minimal T1 and T4 pairs with different lexical properties. Due to the difficulty of finding sufficient stimuli for low lexical frequency (LF) with low homophone neighborhood density (LD), no such stimuli were used in the experimental design. As a result, the stimulus sets consist of high lexical frequency (HF) and low lexical frequency (LF) syllables with high homophone neighborhood density (HD). The lexical frequency in HD and LD syllables always had HF. In total, 90 syllables were selected that had four lexical conditions: HF, LF, HD, and LD. Each lexical condition had 30 syllables, with 15 T1 and 15 T4 syllables. We used the corpus of spoken Chinese based on film subtitles by Cai and Brysbaert (2010). According to the corpus, which is based on 33,546,516 words, the logged frequency^1^ of the high-frequency monosyllabic stimuli (HF) in this experiment is between 2.81 and 4.9 per million, million, with an average of 3.82. The logged frequency of the low-frequency monosyllabic stimuli (LF) is between 0.6 and 2.0, with an average of 1.55.

We used the Modern Chinese Dictionary (5th Version; The Commercial Press) to calculate homophone neighborhood density. There is no standard criterion in the literature on the specific number of homophones to define HD vs. LD. Chen et al. (2009) defined characters with more than seven homophone mates as HD, while those with fewer homophone mates as LD. Wang et al. (2012) used a threshold of nine for HD and two to eight for LD words. Yip (2002), however, has a lower threshold (i.e., six homophone mates) for HD and two for LD. In our stimuli set, we strived for the right balance between HD and LD stimuli (30 for each): for HD syllables, their number of homophone mates is above seven (and up to 84); for LD syllables, their number of homophone mates is below six (and above two). The average number of homophones for the HD and LD syllables is 19 and 4, respectively.^2^ Take “班 /ban1/” (Tones 1–4 are denoted by digits) and “猜 /cai1/” as examples. The HD syllable “班 /ban1/” has 11 homophones, including 般 (‘type’), 颁 (‘to issue’), 斑 (‘spot’), and 搬 (‘to move’). They share the same segmental syllables and tones /ban1/ but are represented by different Chinese characters. The LD syllable “猜 /cai1/” has only one homophone, “偲 /cai1/.” Moreover, “班 /ban1/” and “猜/cai1/” are both high-frequency syllables, so “班 /ban1/” was chosen as an HF syllable with HD and “猜 /cai1/” as an HF syllable with LD.

### Procedure

Participants were exposed to a learning phase formed of four trials using frequent syllables (not used in the test) to familiarize them with the experimental procedure before the test. The 90 target syllables were divided into six blocks, and each block was composed of 15 trials. In the test phase, participants took a self-paced break between the blocks. The order of the trials was randomized for each participant. There was no repetition in any of the trials.

The experiment was conducted in E-prime 2.0 run on a laptop equipped with a Creative SBX-FI5.1 pro sound card. Participants were placed in a quiet room and were asked to read the stimuli in Chinese characters on the computer screen. The responses were recorded using a condenser microphone, and the recordings were stored directly on the computer’s hard disk.

### Data preparation

The acoustic analysis of all data was conducted in Praat (Boersma and Weenink, 2017). All the sound files were manually segmented. The onset and offset of target vowels or tone-bearing syllable rhymes (i.e., vowel or vowel with a nasal coda) determined the time intervals for extracting duration and F0 values. F0 values were sampled at 20 equidistant measurement points using a Praat script. F0 values were converted to speaker-specific z-scores to reduce cross-speaker variability for plotting and statistical analysis. Following Rose (1987), z-scores were calculated with F0*^Zscore^* = (*x_*i*_* – *m)/s*, where *m* is the mean value of x_*i*_ and *s* is the standard deviation, calculated per speaker.

#### Analysis of duration

The duration of T1- and T4-bearing syllable rhymes were modeled using linear mixed-effects (LME) in R (R Core Team, 2015), lme4 (Bates et al., 2014), and lmerTest (Kuznetsova et al., 2017). For the analysis of the effect of lexical frequency, the final model included the following fixed effects: generation (middle-aged vs. young), lexical frequency (HF vs. LF), and tone (T1 vs. T4) (without interaction). The random effects included by-subject slopes for the effects of lexical frequency and tone (without interaction) and by-item intercept. For homophone neighborhood density, the final model included the fixed effects of generation (middle-aged vs. young), homophone neighborhood density (HD vs. LD), and tone (T1 vs. T4) (without interaction). Also, the random effects included by-subject slopes for the effect of homophone neighborhood density and tone (without interaction) and by-item intercept. Note that for both analyses, the fixed factors were added stepwise, and their effects and interactions on model fits were evaluated *via* model comparisons based on log-likelihood ratios. The estimate (*β*), standard error *(SE)*, and *t*-values are reported below.

#### Analysis of F0

Functional data analysis (FDA) (Ramsay et al., 2009a; Gubian et al., 2015) was used to analyze the F0 values. FDA provides a method for analyzing a dataset that consists of entire curves with different durations. Two main procedures were conducted: smoothing with a linear time registration and a Functional Principal Component Analysis (FPCA). Smoothing (with a roughness penalty) was realized by the B-spines (De Boor, 2001). A linear time registration scaled all the smoothed curves into a normalized duration (i.e., 1), used for further FPCA analysis. FPCA provided a model for approximating the (normalized) smoothed curves using the mean curve and a number of Principal Component (PC) curves and their weights (PC scores), based on the formula $\text{f}\left(\text{t}\right)\approx \mu \;(\text{t})+\sum_{\text{j}=1}^{\propto }{\text{s}}_{\text{j}}*{\text{PC}}_{\text{j}}\;(\text{t})$. Here, μ(t) is the mean curve, s_j_ is the PC score (PCs) and PC_j_(t) is the corresponding PC curve.

All FDA was carried out in the FDA R package (Ramsay et al., 2009b). Apart from F0 curves, their instantaneous velocity profiles (which indicate the declining speed of the two falling tones) also reflect dynamic lexical tone articulation (Gauthier et al., 2007; Cheng et al., 2014). Therefore, both the F0 curves and the velocity profiles of the F0 values with the FDA were analyzed. A functional *t*-test was used to calculate the absolute value of the t-statistic at each sampling point of the (normalized) smoothed curves (Ramsay et al., 2009a). A functional *t*-test extends the rationale of the well-known *t*-test and can compare the means of two groups’ curves within specific time domains. Moreover, the first two PC scores (s_1_ and s_2_), which represent most of the variation of the smoothed curves, were further used for performing LME modeling.

Different models were constructed for lexical frequency and homophone neighborhood density effects. There were four lexical frequency effect models for s_1_ and s_2_ in F0 curves and F0 velocity profiles, respectively. The final models included the following fixed effects: generation, lexical frequency and tone (with interaction). The random effects included by-subject slopes for the effects of lexical frequency and tone (with interaction) and by-item intercept. There were also four models for s_1_ and s_2_ in F0 curves and F0 velocity profiles for the homophone neighborhood density effect, respectively. The final models included the following fixed effects: generation, Homophone Neighborhood Density (HND), and tone (with interaction). The random effects included by-subject slopes for the effects of HND and tone (with interaction) and the by-item intercept. Following the advice of one reviewer, we also checked the model constructions with the function (model. selection) in the developed library MuMIn in R (Barton, 2009). Results confirmed that the final model constructions (specified previously) for rhyme duration and F0 were the best considering the AICs weights.

## Results

### Duration of the T1- and T4-carrying syllables

**FIGURE 2:**
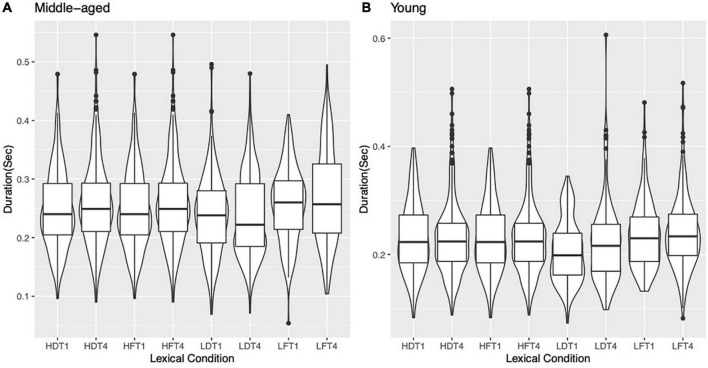
Duration of the rhyme part of syllables for T1 and T4 produced by **(A)** 30 middle-aged participants and **(B)** 30 young participants in the four lexical conditions (HF, LF, HD, and LD). For example, HFT1 represents syllables with high lexical frequency in T1.

Figures 2A,B show the violin plot of the duration of the rhyme part of syllables for T1- and T4 in the four lexical conditions (HF, LF, HD, and LD) produced by middle-aged participants and young participants. For the model of lexical frequency, there was a significant main effect of generation (β = 0.25, *SE* = 0.003, *t* = −10^***^), while for the homophone neighborhood density model, there was a significant main effect of generation (β = −0.03, *SE* = 0.003, *t* = −9.9^***^) and homophone neighborhood density (β = −0.02, *SE* = 0.006, *t* = −2.6*), which we will examine further in Section “Effects of homophone neighborhood density.” It is important to note here that in both models, the lexical tone was not a significant main effect, suggesting the neutralization of T1 and T4 concerning the duration of their tone-bearing syllables.

### F0 curves and velocity profiles

#### Functional data analysis of T1 and T4 F0 contours

**FIGURE 3:**
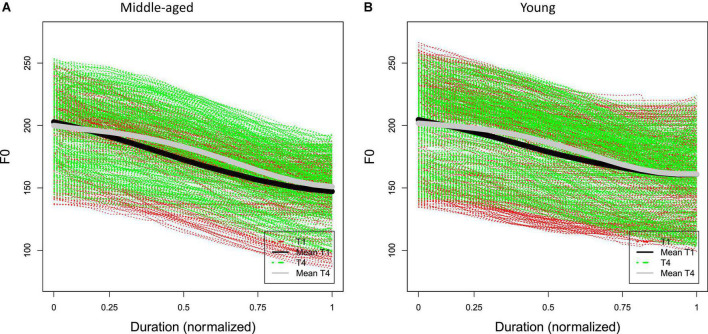
Time-normalized raw F0 contours of T1 and T4 of **(A)** 30 middle-aged participants and **(B)** 30 young participants.

**FIGURE 4:**
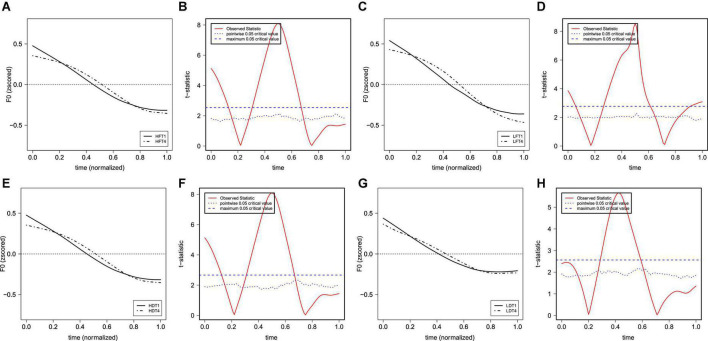
The average of the (normalized) F0 curves for T1 and T4 in the four lexical conditions produced by young participants [**(A)** HF; **(C)** LF; **(E)** HD; **(G)** LD] and their functional *t*-test statistic [**(B)** HF; **(D)** LF; **(F)** HD; **(H)** LD]. The solid lines represent the F0 curves of T1, and the dot-dash lines represent the F0 curves of T4.

**FIGURE 5:**
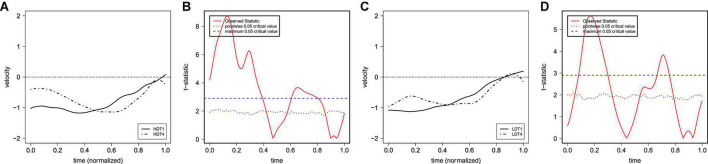
The average of the (normalized) F0 velocity profiles for T1 and T4 in the HD and LD lexical conditions produced by young participants [**(A)** HD; **(C)** LD] and their functional *t*-test statistic [**(B)** HD; **(D)** LD]. The solid lines represent the F0 curves of T1, and the dot-dash lines represent the F0 curves of T4.

Figures 3A,B show the raw F0 contours of T1 and T4 with a normalized duration from middle-aged participants and young participants. The figures show that both T1 and T4 have similar falling F0 contours and ranges (between about 140 and 260 Hz) in both generations. Based on the results of FDA, Figure 4 shows the average F0 curves for T1 and T4 in the four lexical conditions and the results of a between-participant functional *t*-test for the young participants. Figure 5 shows the average of the F0 velocity profiles for T1, and T4 in the HD and LD lexical conditions produced by young participants and their functional *t*-test statistics. For the middle-aged participants, the results were similar but are not shown in this paper. In Figures 4, 5, dotted lines represent the 0.05 critical values for the t-statistic. The higher statistic represents a more conservative critical value.

Although insights into the mean F0 curves and F0 velocity profiles of T1 and T4-based functional *t*-tests were achieved, it is important to note that functional *t*-test only considers the t-statistic of each sampling point in the smoothed curves. Therefore, the LME modeling was employed for the two principal components (PC) scores (s_1_ and s_2_) to investigate further the T1 and T4 F0 contours (curves and velocity profiles). In addition, LME modeling allows for variations due to individual speakers and stimulus items to be taken into account for a greater understanding of the neutralization of the two tones.

#### Linear mixed-effects modeling of principal component scores

**FIGURE 6:**
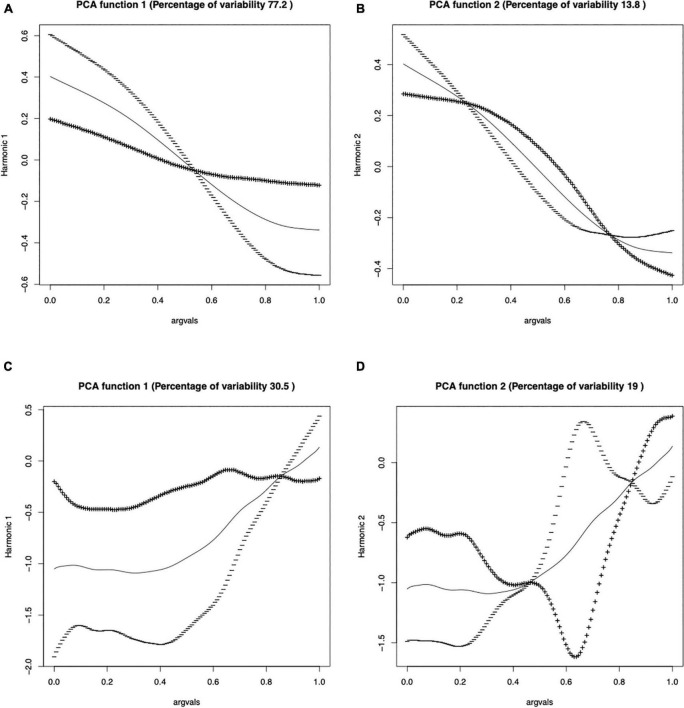
The results of FPCA for **(A)** PC1 (t) and **(B)** PC2 (t) of T1 for F0 curves [**(A)** s_1_; **(B)** s_2_] and F0 velocity profiles [**(C)** s_1_; **(D)** s_2_] in the HF lexical condition for the young participants.

We used FPCA to analyze PC scores. To show how FPCA works, the FPCA results of T1 for F0 curves and F0 velocity profiles in the HF lexical condition for the young participants were plotted in Figure 6. Each panel shown in the solid line is the mean curve μ(t). The ± curves were obtained by adding to or subtracting from μ(t) the curves (a) σ(s_1_) * PC_1_(t) and (b) σ(s_2_) * PC_2_(t). σ denotes standard deviation. PCs are numbered from 1 onwards, and the rank reflects the decreasing percentage of variance in the input data that can be explained by the PCs. As shown in Figure 6, for F0 curves (Figures 6A,B), the FPCA outputs indicate that s_1_ and s_2_ could explain the most variation in the HF lexical condition (77.2 and 13.8%, respectively). Figure 6A suggests that PC1 (s_1_) mainly alters the slope of the F0 curves. Figure 6B suggests that PC2 (s_2_) altered the turning point of the curves. The interpretations of s_1_ and s_2_ are consistent with Gubian (2011). The same goes for the instantaneous F0 velocity profiles (given by the slope of F0 at a single point in time), which indicates the declining speed of the two falling tones. s_1_, s_2_, s_3_, s_4,_ and s_5_ could explain 30.5, 19, 17.2, 9.8, and 7.8% of the variation, respectively. Like the F0 curves, the s_1_ and s_2_ of the velocity profile, which account for more variance, were analyzed (Figures 6C,D). Figure 6C suggests that PC1 (s_1_) mainly alters the slope of the F0 velocity profiles. Figure 6D suggests that PC2 (s_2_) altered the turning point of the velocity profiles. The PC scores enable us to conduct further quantitative analysis of the effect of the two lexical factors on tonal production using LME modeling with s_1_ and s_2_.

We performed LME modeling with s_1_ (indicating the slope) and s_2_ (indicating the turning point) for F0 curves and F0 velocity profiles (i.e., the declining speed of two falling tones). Additionally, different models were fitted for lexical frequency and homophone density effects and the factors of speaker generation and lexical tonal identity. The significant results are presented in Tables 1, 2.

**TABLE 1:** Summary of linear mixed-effects modeling for F0 curves.

Lexical condition	PCs	Fixed effects	β	*SE*	*df*	*t*	*p*
Lexical frequency	s_1_	Generation × Tone	0.04	0.01	3862	3.5	***
	s_2_	Lexical frequency	0.05	0.01	48	4.7	***
	s_2_	Tone	0.05	0.006	83	7.4	***
	s_2_	Generation × Lexical frequency	–0.05	0.008	2364	–6.8	***
	s_2_	Lexical frequency × Tone	–0.09	0.01	38	–6.4	***
	s_2_	Generation × Tone × Lexical frequency	0.1	0.01	3255	9.1	***
Homophone neighborhood density	s_1_	Generation × Tone	0.04	0.01	3175	3.1	**
	s_2_	Tone	0.05	0.008	69.21	5.9	***
	s_2_	Generation × Homophone neighborhood density	0.02	0.007	1752	2.5	*
	s_2_	Generation × Tone × Homophone neighborhood density	–0.02	0.009	1471	–2.3	*

*p < 0.05, **p < 0.01, ***p < 0.001.

**TABLE 2:** Summary of linear mixed-effects modeling for F0 velocity profiles.

Lexical condition	PCs	Fixed effects	β	*SE*	*df*	*t*	*p*
Lexical frequency	s_2_	Lexical frequency × Tone	–0.18	0.08	102	–2.2	*
Homophone neighborhood density	s_1_	Generation × Homophone neighborhood density	–0.13	0.06	2571	–3.0	**
	s_1_	Generation × Tone × Homophone neighborhood density	0.25	0.08	2580	3.1	**
	s_2_	Homophone neighborhood density	–0.15	0.05	77	–3.1	**
	s_2_	Homophone neighborhood density × Tone	0.24	0.07	78	3.5	***
	s_2_	Generation × Tone × Homophone neighborhood density	–0.15	0.07	1711	–2.1	*

*p < 0.05, **p < 0.01, ***p < 0.001.

For F0 curves (Table 1), the dominant effect for s_1_ lies in the interaction of speaker generation and tonal identity. Further details of the interaction are discussed in Section “Generational differences in the F0 characteristics of T1 and T4.” For s_2_, there were significant three-way interactions (Generation × Tone × Lexical frequency and Generation × Tone × Homophone neighborhood density). Therefore, separate models from the subset data about generation, tone, and lexical conditions (lexical frequency and homophone neighborhood density) were run to reveal the differences between the two falling tones for each lexical condition in each generation. The significant results are presented in Table 3.

**TABLE 3:** Summary of linear mixed-effects modeling for F0 curves (from subset data of generation, tone, and lexical conditions).

Generation	Lexical condition	Tone	PCs	β	*SE*	*df*	*t*	*p*
Middle	HF	T1 vs. T4	s_2_	0.04	0.002	53	6.4	***
	LF			–0.04	0.01	39	–2.6	*
	HD			0.04	0.009	51	4.6	***
	LD			0.05	0.01	34	4.0	***
Young	HF			0.05	0.007	53	6.4	***
	LF			0.05	0.01	34	3.7	***
	HD			0.06	0.009	38	6.2	***
	LD			0.03	0.009	29	3.7	***

*p < 0.05, **p < 0.01, ***p < 0.001.

From Table 3, significant differences between T1 and T4 in s_2_ (corresponding to the turning point of F0 curves) can be seen for all four lexical conditions in middle-aged and young participants. Specifically, the turning point for T4 was earlier than that for T1 across generations and lexical conditions. There were also significant two-way interactions for the s_2_ of F0 curves for speaker generation and lexical frequency, lexical frequency and tonal identity, and speaker generation and homophone neighborhood density. Separate models from subset data were also run to reveal the differences. Results showed that the F0 turning point of syllables with high lexical frequency was earlier than syllables with low lexical frequency for T1. However, for T4, the F0 turning point of syllables with low lexical frequency was earlier than syllables with high lexical frequency. This raises questions regarding the exact effect of lexical frequency on tonal realization. Further details of the interaction between speaker generation and homophone neighborhood density are reported in Section “Effects of homophone neighborhood density.”

For F0 velocity profiles (Table 2), we observe a significant interaction of lexical frequency and tone for s_2_. This means that there were significant differences in the F0’s turning point between the two falling tones in different lexical frequencies. The pattern is similar to the contradictory findings of F0 curves in T1 and T4. Homophone neighborhood density, on the other hand, showed significant three-way interactions with both Generation and Tone for both the s_1_ and s_2_ of the F0 velocity profiles. Separate models from the subset data of generation, tone, and homophone neighborhood density were conducted. The main significant results for the models are presented in Table 4.

**TABLE 4:** Summary of linear mixed-effects modeling for F0 velocity profiles (from subset data of generation, tone, and homophone neighborhood density).

Generation	Lexical condition	Tone	PCs	β	*SE*	*df*	*t*	*p*
Middle	LD	T1 vs. T4	s_1_	–0.25	0.09	37	–2.8	**
			s_2_	0.18	0.07	32	2.5	*
Young			s_2_	0.25	0.09	15	2.8	**

*p < 0.05, **p < 0.01, ***p < 0.001.

Table 4 shows a significant difference in T1 and T4 for s_1_ in the LD lexical condition among the middle-aged participants. The slope of the F0 velocity profiles for T4 was steeper than those for T1. For s_2_, T1 and T4 differed again only in LD, but for both middle-aged and young participants, the turning point of T4 was earlier.

#### Generational differences in the F0 characteristics of T1 and T4

**FIGURE 7:**
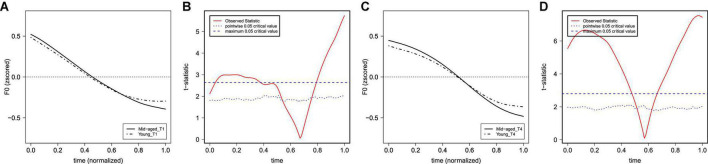
The average of the (normalized) F0 curves between the middle-aged and young participants for T1 **(A)** and T4 **(C)** and their functional *t*-test statistics in panels **(B,D)**. The solid lines represent the F0 curves of middle-aged participants, and the dot-dash lines represent the F0 curves of young participants.

This section further reports the details of the incomplete neutralization of T1 and T4 by comparing how speakers of the two generations produce each of the two tones. The specific direction of the sound changes in T1 and T4 can also be explored. Note that for F0 curves, the dominant effect lies in the significant interaction of Generation and Tone of s_1_, regardless of lexical frequency and neighborhood density. Figure 7 shows the average F0 curves of T1 and T4 and the results of functional *t*-tests between the two generations. There were significant differences in the initial and later parts of the F0 curves between the two generations in the production of both T1 and T4. Results of the LME modeling (Table 5) showed a significant difference between middle-aged speakers and young speakers for T1 and T4. Specifically, the slope (indexed *via* the s_1_ of F0 curves) of both T1 and T4 was steeper for young participants than the one for middle-aged participants.

**TABLE 5:** Summary of linear mixed-effects modeling for F0 curves.

Generation	F0	Tone	PCs	β	*SE*	*df*	*t*	*p*
Middle vs. Young	Curves	T1	s_1_	0.04	0.01	32	3.6	**
		T4		0.07	0.03	30	2.8	**

*p < 0.05, **p < 0.01, ***p < 0.001.

#### Effects of homophone neighborhood density

As stated previously, a key effect of homophone neighborhood density was found on the rhyme duration of the Tone 1- and Tone 4-carrying syllables. Furthermore, homophone neighborhood density also interacted significantly with generation and tone. These findings indicate the effect of homophone neighborhood density on lexical production, which has not been reported in the literature. Therefore, this factor will be detailed further in the proceeding section.

First, LME modeling was performed with syllable rhyme duration as the dependent variable and homophone neighborhood density as an independent variable. The by-subject slope for the effect of homophone neighborhood density and the by-item intercept were included as random effects in the final model. For young speakers, the rhyme of T1-carrying syllables showed a significant main effect of homophone neighborhood density (β = −0.02, *SE* = 0.01, *t* = −2.0*), with the duration in LD on average 20 ms shorter than HD. The rhyme duration of T4-carrying syllables was also shorter (15ms) in LD-syllables than HD-syllables (β = −0.015, *SE* = 0.007, *t* = −2.1*). Similar results were found for middle-aged speakers (T1: 17 ms shorter in LD; β = −0.017, *SE* = 0.007, *t* = −2.5*; T4: 14 ms shorter in LD; β = −0.014, *SE* = 0.006, *t* = −2.2*).

Second, LME modeling was performed for the FPCA results of the F0 data based on the significance tests reported earlier. Specifically, we focused on the s_2_ of F0 curves and both s_1_ and s_2_ of F0 velocity profiles. The effect of homophone neighborhood density on T1 and T4 produced by speakers of the two generations was a leading point of interest. Separate models were run with homophone neighborhood density as the fixed effect, while the by-subject and the by-item intercept were included as random effects. Table 6 shows that there was a significant effect of neighborhood density on the s_2_ (i.e., the turning point of F0 curves and F0 velocity profiles) between HD and LD for young speakers. For F0 curves, the turning point for LD was earlier than HD for both T1 and T4. For F0 velocity profiles, the turning point for LD was earlier than HD for T1 but was later than HD for T4. Significant differences were also found in the velocity profiles for the middle-aged speakers (s_2_ in both T1 and T4). The turning point of F0 velocity profiles for LD was earlier than HD for both T1 and T4.

**TABLE 6:** Summary of linear mixed-effects modeling for F0 curves and F0 velocity profiles in the lexical condition of homophone neighborhood density for T1 and T4.

Generation	F0	Lexical condition	Tone	PCs	β	*SE*	*df*	*t*	*p*
Middle	Velocity profiles	HD vs. LD	T1	s_2_	–0.19	0.08	52	–2.2	*
			T4	s_2_	0.12	0.05	52	2.2	*
Young	Curves		T1	s_2_	–0.03	0.008	29	–3.1	**
	Velocity profiles				–0.12	0.06	31	–2.1	*
	Curves		T4	s_2_	–0.03	0.008	29	–4.4	***
	Velocity profiles				0.27	0.07	37	4.0	***

*p < 0.05, **p < 0.01, ***p < 0.001.

## Discussion and conclusion

The current study investigated the acoustic realizations of two falling tones (i.e., T1 and T4) in Dalian Mandarin. The goal of this study was to understand the effects of (1) lexical properties (i.e., frequency and homophone neighborhood density) and (2) generation of speakers (i.e., middle-aged vs. young) on the neutralization of the two falling tones. Recordings of 45 pairs of T1 to T4 carrying syllables were elicited from 60 native participants who are from two different generations (i.e., middle-aged vs. young). The duration and F0 of the rhyme of the tone-carrying syllables were quantitatively analyzed.

Results showed no significant durational difference between the T1 and T4 tone-bearing syllable rhymes, contrary to the reports by Gao (2007) and Liu (2009). Concerning the F0 contours of the two falling tones, however, we found subtle but statistically significant differences (Figure 3), reflected in terms of both the F0 curves (Figure 4) and F0 velocity profiles (Figure 5) of the tonal F0 contours. Generally speaking, T4 showed a consistently earlier F0 turning point than T1 for both generations of speakers. Although interactions were found among lexical properties, speaker generation, and tonal identity on the duration and F0 patterns of the two lexical tones (as discussed further below), our results confirmed that the neutralization of T1 and T4 is not complete. The general pattern of incomplete neutralization between T1 and T4 has remained rather stable across the middle-aged and young speakers.

Significant three-way interactions were found for lexical frequency, speaker generation, and tone (Table 1). Significant differences between T1 and T4 were found regardless of the frequency of the tone-carrying syllables and for both generations of speakers (Table 3). This suggests little effect of lexical frequency on the neutralization of T1–T4. Two-way interactions were also found between lexical frequency and tonal identity for the s_2_ (turning point) of F0 curves and F0 velocity profiles (Tables 1, 2). For T1, the F0 turning point was earlier in syllables with high lexical frequency. For T4, the F0 turning point was earlier in syllables with low lexical frequency. It is not clear why frequency showed different effects on T1 and T4 tonal realization. Future research is needed to confirm and understand this effect.

For homophone neighborhood density, there were significant three-way interactions with speaker generation and tone (as shown in Tables 1, 2). In the low homophone neighborhood density condition, T4 showed a steeper falling slope than T1 (reflected in the s_1_ of the velocity profiles) for the middle-aged speakers. Furthermore, T4 showed an earlier turning point (reflected in the s_2_ of the velocity profiles) for both generations of speakers (Table 4). Focusing on individual tonal production, it was found that tones carried by syllables with low homophone neighborhood density were hyper-articulated with a longer duration than that with high homophone neighborhood density. There was also a significant effect of homophone neighborhood density on the s_2_ of the F0 curves and velocity profiles between the high and low homophone neighborhood density conditions, but only for young speakers. Specifically, both T1 and T4 showed an earlier F0 turning point in the low homophone neighborhood density condition (Table 6).

Our results on the effect of speaker generation suggest that the T1 to T4 contrast in Dalian Mandarin (or their incomplete neutralization) is rather stable across the two generations. However, the different generations of speakers who took part in this study did show some differences in the specific F0 contours of the two lexical tones, suggesting ongoing changes in the F0 realization of both T1 and T4. The dominant effect lies in the significant interaction of Generation and Tone for the slope of F0 curves (s_1_), regardless of lexical frequency and neighborhood density. As shown in Figure 7 and Table 5, the slope of F0 curves for both T1 and T4 were steeper for young participants than for middle-aged participants.

It is seen in the literature that a large number of speakers of Dalian mandarin are descendants of migrants from Shandong Province, whose native dialects (spoken in, e.g., Weihai and Yantai City) have falling F0 contours for both T1 and T4 cognates. It is generally accepted that the contact of these emigrants with local Dalian Mandarin speakers around 1904 (the Twentieth Century) initiated the merger between T1 and T4. Such language-contact-induced sound change is in line with the social variations and sound changes charted out in Labov (2001, 2011). In Gao (2007), we know that in Dalian Mandarin, the citation form of T1 was changed from 312 in speakers of age between 70 and 80 (old-aged speakers) to 411 in speakers of age between 50 and 59 (middle-aged speakers), and then from 411 to 51 of age below 29 (young speakers). For T4, it was changed from 53 in old-aged speakers to 52 in middle-aged speakers, and then from 52 to 51 in young speakers. In the participants of this study, both the middle-aged (mean age: 50; SD: 3.6) and young speakers (mean age: 22; SD: 3.6) realized the two tones as a falling tone (51). This suggests that the merger of the T1 and T4 has been rather stable among our two generations of speakers. From the reports in the existing literature and our data, it can be suggested that the two falling tones have neutralized gradually by approximation. That is, it is the gradual approximation of both the T1 and T4 tonal targets that has resulted in the merger of the two tones into the same falling tone contour (51), and the changes have been rather symmetrical. Garrett and Johnson (2013), however, reported contradictory results and found that sound change is typically directional and asymmetric in speech production and perception.

It is important to note that the subtle differences between T1 and T4 have remained stable across the two generations of our speakers. Given the lack of findings on the effect of speaker generation on the contrast between T1 and T4, we may conclude that the sound change reported in the literature (Gao, 2007; Liu, 2009) has already arrived at the state of incomplete neutralization among our middle-aged speakers. This suggests that while the merging of T1 and T4 has been completed by the time of their studies, the nature of the merger is incomplete neutralization. Languages, however, continually evolve, and this is also true for the two incompletely neutralized tones. Changes were observed in both T1 and T4 from the middle-aged to the young-generation speakers. In particular, the younger speakers produced both T1 and T4 with steeper F0 curves than the middle-aged speakers. Our pilot data also suggest that the acoustic features of T4 in Dalian Mandarin are similar to that of T4 in Standard Mandarin. Furthermore, native speakers of Dalian Mandarin are not able to tell the difference between the two falling tones. The change in citation form from 53 to 51 for T4 in Dalian Mandarin is probably due to the language contact with Standard Mandarin, especially for speakers from the young generation. Nevertheless, the way T1 has changed cannot be attributed to merely the influence of Standard Mandarin; otherwise, T1 and T4 should have become more different instead of being incompletely neutralized. It can be speculated that T1 became closer to the T4 instead of being closer to the T1 of Standard Mandarin because of the regional identity of the young generation. It is quite well-known that the characteristic of T1, a falling tendency, marks the regional identity of the local residents. It is likely that, speakers have therefore preferred to keep the falling F0 pattern of T1 instead of adjusting T1 to a high-level tone (as in Standard Mandarin). Future research is needed with, for example, questionnaires and interviews to verify speakers’ regional identity and their preference for tonal acoustic realization.

Various factors have been discussed in the literature that condition sound changes. The effects of lexical frequency and homophone neighborhood density have been investigated in the current study. Results showed that the degree of T1–T4 neutralization does not vary as a function of lexical frequency. Specifically, no significant differences between the two falling tones were found for different lexical frequencies. In the literature on the role of lexical frequency in sound change, two different mechanisms have been posted: articulatorily-motivated and analogical changes. Articulatorily-motivated change typically affects high-frequency words first, while analogical sound change affects low-frequency words first (Phillips, 1984; Bybee, 2007). The effect of lexical frequency may also vary depending on the stage of sound change, namely, whether it is in progress or stable. For non-tonal languages, it has been claimed that the effect of lexical frequency could be the largest when the change is in progress and the smallest when the change has reached a stationary stage (Hay et al., 2015). In the case of Cantonese, which has tonal near mergers, Mok et al. (2013) found that word frequency had little impact. Cantonese tone mergers are assumed to be relatively stable. The current study echoes the findings in Cantonese and confirms the lack of lexical-frequency effect in the neutralization of tones at a relatively stable stage.

Frequency showed an effect on the acoustic realization of the individual lexical tones and their tone-carrying syllables. Specifically, for T1, the F0 turning point was found to be earlier in syllables with high lexical frequency, but for T4, the F0 turning point was earlier in syllables with low lexical frequency. This is a pattern that must be further replicated before any meaningful discussion can occur.

For the effect of homophone neighborhood density, results showed that with low homophone neighborhood density, we could observe a significant difference between the two falling tones. The slope of the F0 falling was found to be steeper in T4 than in T1. The current study is the first to examine the effect of homophone neighborhood density on tonal neutralization. Our results suggest that tones in syllables with low homophone neighborhood density tend to maintain their contrast, while those with high homophone neighborhood density may have been more completely neutralized. Further research is needed to verify this finding.

When examining homophone neighborhood density, it can be seen that the individual tones were hyper-articulated with a longer duration and a later F0 turning point in syllables with high homophone neighborhood density. To understand the patterns, it may be necessary to employ several models from the literature, namely the Neighborhood Activation Model and the communication-based accounts (e.g., Luce, 1986; Goldinger et al., 1989; Vitevitch and Sommers, 2003; Baus et al., 2008; Taler et al., 2010; Chen and Mirman, 2012; Gahl and Strand, 2016; Yao and Sharma, 2017; Arutiunian and Lopukhina, 2020; Karimi and Diaz, 2020). A crucial assumption of the Neighborhood Activation Model (NAM) is that the activation and inhibition of the target words during speech processing. During the processing of spoken words, the target word and all the other competitors (e.g., neighbors) are activated. NAM was originally proposed to model the results of word recognition. A number of studies, however, have also used the notions of activation and competition of targets and competitors modeled by the NAM to understand speech production. The number of lexical neighbors of a target word (i.e., its neighborhood density) influences the selection and production of the target word. It is easier to produce a target word with more lexical neighbors (dense phonological neighborhood density—more competitors). Typically, speech error rates, naming accuracies, and latencies have been examined to infer the effect of neighborhood density on production. In the current study, we found that the duration for HD syllables was longer than their LD counterparts. This suggests the possibility that there is an inhibitory effect of HD on tonal production. Needless to say, replication studies are needed to verify the findings and this interpretation.

Communication-based accounts support the idea that efficient communication is the main aim of language processing. If a target word is highly similar to its neighbors/competitors, it may generate high communication uncertainty. Thus, the speaker is expected to spend more time and articulatory effort to precisely produce the acoustic signal to increase the probability of it being accurately recognized. Both NAM and communication-based accounts may be related to the theory of Hypo- and Hyper-articulation (H&H) (Lindblom, 1990) in lexical production, which states that speakers produce strengthened phonetic forms when they anticipate perceptual difficulty on the part of their listeners (Buz et al., 2016). In the current study, our stimuli consist of minimal T1 and T4 pairs with different neighborhood densities. T1 and T4 share the same segmental information but with different falling tones, which are close to each other. When the target is T1, its competitors mainly include its neighbors (homophones), which share the same segmental and tonal information, as well as its minimal-paired T4 syllables (and homophone neighbors). The parallel representations of related segments and tones are expected to be activated due to tonal (incomplete) neutralization. Whether homophones and minimal pairs can be hyperarticulated or distinguished with different acoustic characteristics has been under discussion in the existing literature. For example, growing evidence shows that homophones may differ in pronunciation depending on their intended meaning. On the surface, this does not make much sense, as homophones are words that have the same phonological form but distinct meanings. However, the distinctness of the meanings may result in different pronunciations over time. There is some evidence that speakers produce homophones (e.g., bridal vs. bridle) with emotional valence appropriate to the intended meaning, leading to differences in duration and F0. Some function words with multiple meanings may also differ in duration in spontaneous speech depending on the intended meaning (e.g., Nygaard et al., 2002). The meaning of the target word matters and the homophone could be distinguished with different acoustic characteristics. Wedel et al. (2018) studied the phonetic specificity of contrastive hyper-articulation in natural speech considering minimal pairs and their results showed that cue-specific minimal pairs significantly predicted cue hyper-articulation. Therefore, considering all the competitors from meanings, minimal-paired tonal/segmental information, and listeners’ expectation, homophonous targets may be hyperarticulated or realized differently with distinct acoustic characteristics in lexical production. Following these reasons, we may expect that tones with high neighborhood density should better maintain a contrast, which explains why both T1 and T4 with high neighborhood showed longer duration and a later F0 turning point. What we observed, however, is that the T1 and T4 contrast is more reliably detected in syllables with low homophone neighborhood density. Future research is needed to verify this pattern and also to tap into the effect of different ways of defining phonological connectedness (e.g., Kapatsinski, 2006) within a speaker’s mental lexicon and how such networks affect speech production and sound change.

Another crucial notion in relation to the effects of frequency and neighborhood density on tonal merger, argued to condition sound change, is the functional load (FL) (Surendran and Levow, 2004; Oh et al., 2013; Wedel et al., 2013a,b; Vogel et al., 2016). FL has been suggested as an important factor in determining whether two phonemes are merged in a language (Martinet, 1933). Wedel et al. (2013a) reported the first large-scale study of the functional load hypothesis using data from sound changes in eight languages, including English, German, Dutch, and Cantonese. Results showed that the more minimal pairs defined by a phoneme pair, the less likely that phoneme pair is to have merged. Even though Cantonese was used as one of the case languages in that study, the tonal merger between T2 and T5 in Cantonese with different functional loads was not concluded in detail. Note that the way of FL calculation for a target phoneme could not be applied to our current study (due to the lack of comparable corpus). The calculation of FL in Oh et al. (2013) was followed, which focuses on the cross-language comparison of functional load for vowels, consonants, and especially tones. According to Oh et al. (2013), if a target is *pan* in T4 (pan4), its FL computation depends on pan*, i.e., *pan* with different tones. We did a similar computation for our minimal-paired syllables of T1 and T4 and also one based on the database^3^ (Neergaard and Huang, 2019). The results from both methods were consistent; syllables with high neighborhood density also have high FL, while those with low neighborhood density qualify as having low FL. The acoustic realizations of T1 and T4 with low neighborhood density (i.e., low FL), however, showed more differences than those with high neighborhood density (i.e., high FL). This is the opposite pattern from previous studies (Wedel et al., 2013b), where a higher FL was found to lead to a lower likelihood of a merger. Note that previous studies focused on phoneme contrast instead of tonal contrast, and our ways of calculating FL are not exactly the same. How to calculate FL and how exactly FL affects tonal neutralization must therefore be investigated further in order to gain a thorough understanding of FL and sound change.

In conclusion, the two falling tones in Dalian Mandarin are incompletely neutralized. The status of incomplete neutralization is relatively stable across speakers from middle-aged and young generations. Lexical frequency showed little effect on tonal neutralization, and low homophone neighborhood density helped to maintain the incompletely neutralized contrast. The effects of lexical frequency and homophone neighborhood density on tonal acoustic realization have also been investigated. It has been found that while some of the effects are predicted by existing theories of speech production and known mechanisms of sound change, the results raised more questions than they answered in regards to their effects. Further research is evidently needed to replicate these findings and to better understand the effects of lexical properties on tonal production, perception, and change.

## Data availability statement

The raw data supporting the conclusions of this article will be made available by the authors, without undue reservation.

## Ethics statement

The studies involving human participants were reviewed and approved by the Ethics Committee of the Leiden University Centre for Linguistics, Leiden, Netherlands. The patients/participants provided their written informed consent to participate in this study.

## Author contributions

YB and YC designed the experiment and revised the manuscript. YB collected, processed, analyzed the data and wrote the first draft of the manuscript with feedback from YC. Both authors approved the submitted version.

## Footnotes

1 This value is based on log_10_ (FREQcount + 1), in which FREQcount is the number of times the word appears in the corpus.

2 The calculation was also double-checked with data base (http://dowls.site/) generated by Neergaard and Huang (2019).

3
http://dowls.site/

## Supplementary material

Supplementary Table 1 Stimuli for the production experiment.
